# Supplementary material for: Plasmonic Ni-doped W18O49 with dual active sites drives efficient methanol dehydration to dimethyl ether
Source: Nat Commun. 2025 Nov 17;16:10062. doi: 10.1038/s41467-025-65040-3 (PMC12623422; doi:10.1038/s41467-025-65040-3)
Supplement: Supplementary file 1 — Supplementary Information [file 41467_2025_65040_MOESM1_ESM.pdf]

Supplementary Information

Plasmonic Ni-doped $W_{18}O_{49}$ with Dual Active Sites Drives Efficient Methanol Dehydration to Dimethyl Ether

Dehua Tian[1], Yinlan Liang[1], Zhaoke Zheng[2,*], Liang Mao[3], Xiaoyan Cai[3], Yizhen Chen[4], Xiangxian Wang[4], Xiaolei Liu[1,*], Juan Li[1], Zeyan Wang[2], Can Xue[5], Baojun Li[1] and Zaizhu Lou (娄在祝)[1,*]

[1]Guangdong Provincial Key Laboratory of Nanophotonic Manipulation, Institute of Nanophotonics, College of Physics & Optoelectronic Engineering, Jinan University, Guangzhou 511443, China

[2]State Key Laboratory of Crystal Materials, Shandong University, Jinan 250100, China

[3]School of Materials Science and Physics, China University of Mining and Technology, Xuzhou 221116, Jiangsu Province, China

[4]School of Science, Lanzhou University of Technology, Lanzhou 730050, China

[5]School of Materials Science & Engineering, Nanyang Technological University, 639798, Singapore

E-mail: zkzheng@sdu.edu.cn (Z. Zheng); liuxiaolei@jnu.edu.cn (X. Liu); zzlou@jnu.edu.cn (Z. Lou)

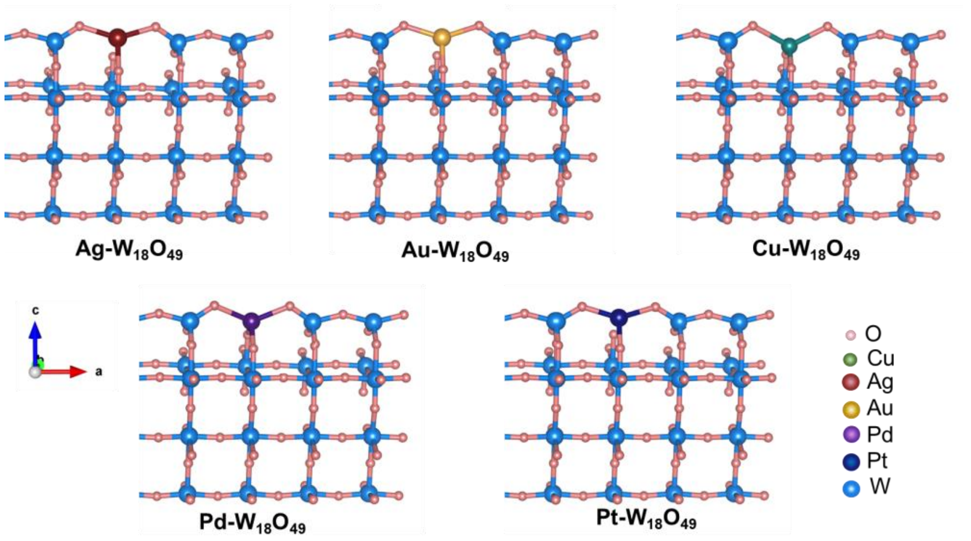

Supplementary Figure 1. Surface structures of various metal-doped $W_{18}O_{49}$ containing Ag, Au, Cu, Pd, and Pt.

Supplementary Table 1. ICP-MS measurement results of Ni_x-$W_{18}O_{49}$

Sample	m_0 (g)	C_o (μg/L)	f	C_1 (μg/L)	C_x (μg/kg)	W (%)
$Ni_{0.15}$-$W_{18}O_{49}$	0.02050	30.1117	20	602	1468864.97	0.15%
	0.02101	31.3104	20	626	1490263.06	
$Ni_{0.32}$-$W_{18}O_{49}$	0.02000	62.9644	20	1259	3148217.62	0.32%
	0.02005	66.5605	20	1331	3319727.00	
$Ni_{0.66}$-$W_{18}O_{49}$	0.02016	26.7206	100	2672	6627144.22	0.66%
	0.02041	27.0748	100	2707	6632734.43	
$Ni_{1.0}$-$W_{18}O_{49}$	0.02102	43.4853	100	4348	10343797.56	1.0%
	0.02103	42.5688	100	4257	10120964.10	
$Ni_{1.4}$-$W_{18}O_{49}$	0.02112	58.3967	100	5840	13824984.45	1.4%
	0.02081	56.6844	100	5668	13619518.16	

m_0: Sample weight; V_0: 50 mL; C_o: Element concentration in the test solution; f: Dilution factor; C_1: Element concentration in the original digestion solution; C_x: Elemental content of the sample; W (%): The final test result of the measured element is expressed as a percentage of the total composition. The test result is obtained using the following formula:

$$C_x(\mu g/kg) = \frac{C_0(\mu g/L) \times f \times V_0(mL) \times 10^{-3}}{m(g) \times 10^{-3}} = \frac{C_1(\mu g/L) \times V_0(mL) \times 10^{-3}}{m(g) \times 10^{-3}}$$

$$W(\%) = \frac{C_x(\mu g/kg)}{10^9}$$

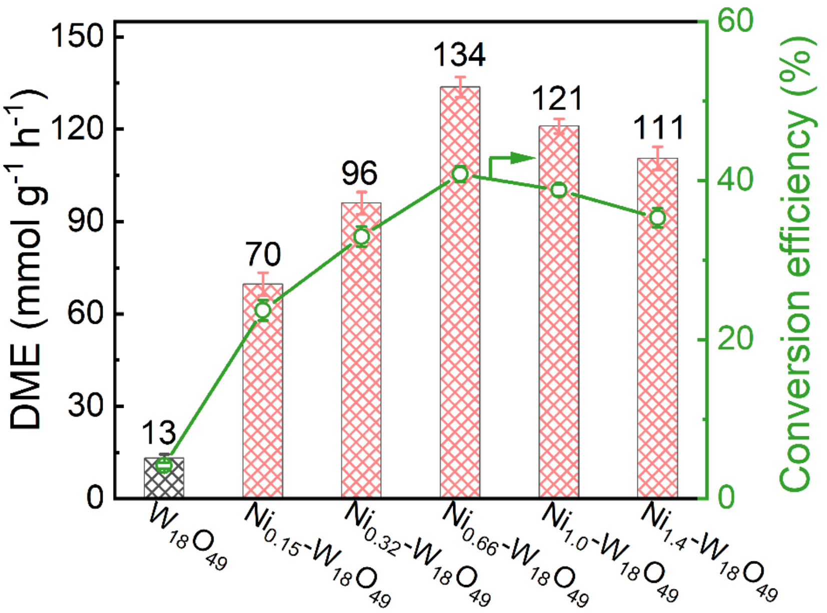

Supplementary Figure 2. Photocatalytic DME generation rate and methanol conversion efficiency over catalysts $W_{18}O_{49}$ and Ni_x-$W_{18}O_{49}$. Error bars correspond to the standard deviation determined from three independent measurements.

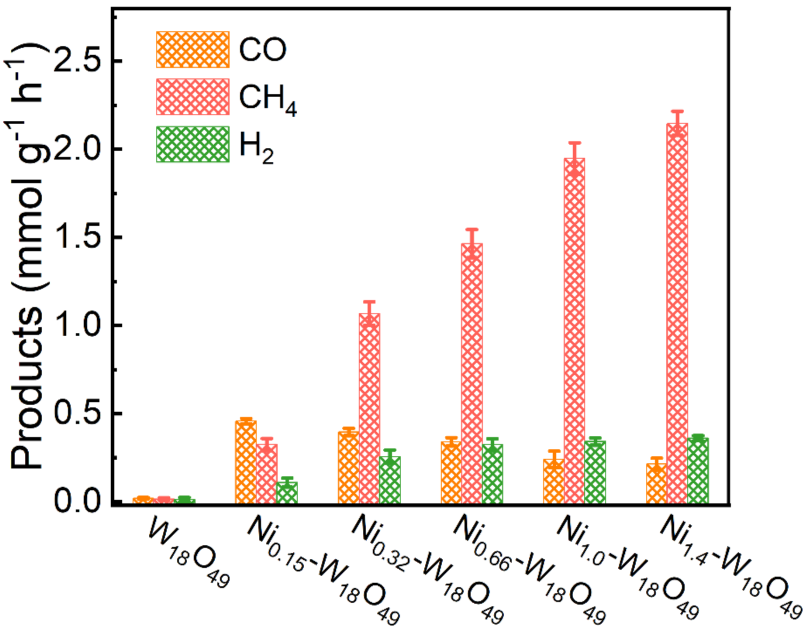

Supplementary Figure 3. Generation rates of H_2, CH_4, and CO during methanol dehydration reaction over $W_{18}O_{49}$ and Ni_x-$W_{18}O_{49}$. Error bars correspond to the standard deviation determined from three independent measurements.

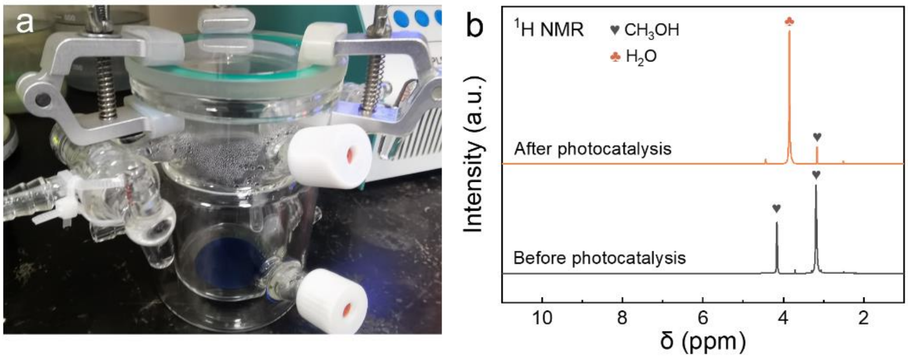

Supplementary Figure 4. a Figure of the reactor after photocatalytic reaction. **b** 1H NMR spectra of liquids before and after photocatalysis.

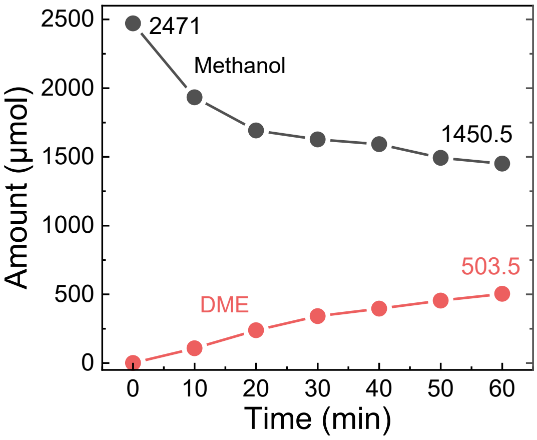

Supplementary Figure 5. The reduction of methanol and the generation of DME during photocatalytic reaction.

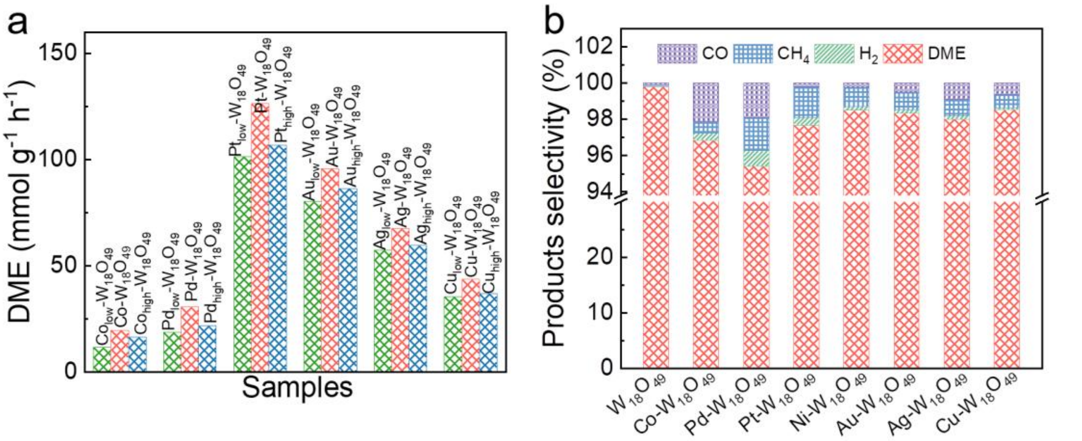

Supplementary Figure 6. a Photocatalytic DME generation rates over Co_{low}-$W_{18}O_{49}$, Co-$W_{18}O_{49}$, Co_{high}-$W_{18}O_{49}$, Pd_{low}-$W_{18}O_{49}$, Pd-$W_{18}O_{49}$, Pd_{high}-$W_{18}O_{49}$, Pt_{low}-$W_{18}O_{49}$, Pt-$W_{18}O_{49}$, Pt_{high}-$W_{18}O_{49}$, Au_{low}-$W_{18}O_{49}$, Au-$W_{18}O_{49}$, Au_{high}-$W_{18}O_{49}$, Ag_{low}-$W_{18}O_{49}$, Ag-$W_{18}O_{49}$, Ag_{high}-$W_{18}O_{49}$, Cu_{low}-$W_{18}O_{49}$, Cu-$W_{18}O_{49}$, and Cu_{high}-$W_{18}O_{49}$, respectively. **b** Product selectivity of photocatalytic methanol dehydration over $W_{18}O_{49}$, Co-$W_{18}O_{49}$,

$Pd\text{-}W_{18}O_{49}$, $Pt\text{-}W_{18}O_{49}$, $Au\text{-}W_{18}O_{49}$, $Ag\text{-}W_{18}O_{49}$ and $Cu\text{-}W_{18}O_{49}$, respectively. The measurements were only performed once.

Supplementary Table 2. ICP-MS measurement results of $M\text{-}W_{18}O_{49}$ (M= Co, Pd, Pt, Au, Ag, and Cu)

Sample	m_0 (g)	Element	C_o (mg/L)	f	C_1 (mg/L)	C_x (mg/kg)	W (%)
$Au\text{-}W_{18}O_{49}$	0.03077	Au	5.802	1	5.802	9428.01	0.963%
	0.03098		6.0873	1	6.0873	9824.57	
$Ag\text{-}W_{18}O_{49}$	0.03041	Ag	4.5307	1	4.5307	7449.43	0.746%
	0.03138		4.6914	1	4.6914	7475.16	
$Cu\text{-}W_{18}O_{49}$	0.03119	Cu	4.2723	1	4.2723	6848.61	0.687%
	0.03114		4.2864	1	4.2864	6882.51	
$Pt\text{-}W_{18}O_{49}$	0.03084	Pt	1.2773	5	6.3865	10354.25	1.04%
	0.03071		1.2764	5	6.382	10390.75	
$Pd\text{-}W_{18}O_{49}$	0.0305	Pd	0.8954	5	4.477	7339.34	0.73%
	0.03129		0.9145	5	4.5725	7306.65	
$Co\text{-}W_{18}O_{49}$	0.03108	Co	3.7787	1	3.7787	6078.96	0.61%
	0.03082		3.7654	1	3.7654	6108.64	

m_0: Sample weight; V_0: 50 mL; C_o: Element concentration in the test solution; f: Dilution factor; C_1: Element concentration in the original digestion solution; C_x: Elemental content of the sample; W (%): The final test result of the measured element is expressed as a percentage of the total composition. The test result is obtained using the following formula:

$$C_x(\mu g/kg) = \frac{C_0(\mu g/L) \times f \times V_0(mL) \times 10^{-3}}{m(g) \times 10^{-3}} = \frac{C_1(mg/L) \times V_0(mL)}{m(g) \times 10^{-3}}$$

$$W(\%) = \frac{C_x(mg/kg)}{10^6}$$

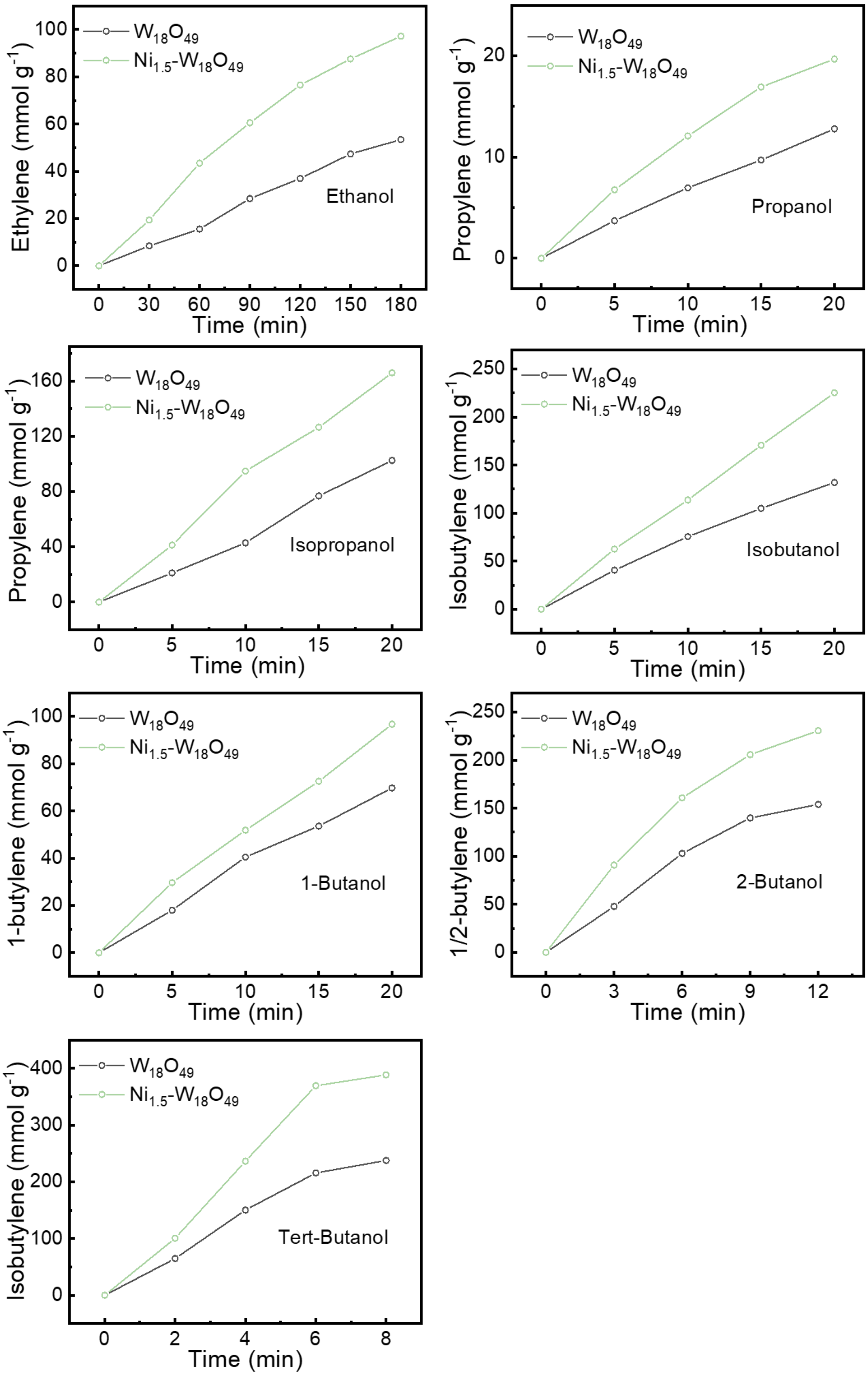

Supplementary Figure 7. Photocatalytic dehydration of other alcohols (C2, C3, and C4) over $Ni_{0.66}$-$W_{18}O_{49}$ for alkene generation. Light source: 400 mW cm^{-2}, AM 1.5G irradiation. The measurements were only performed once.

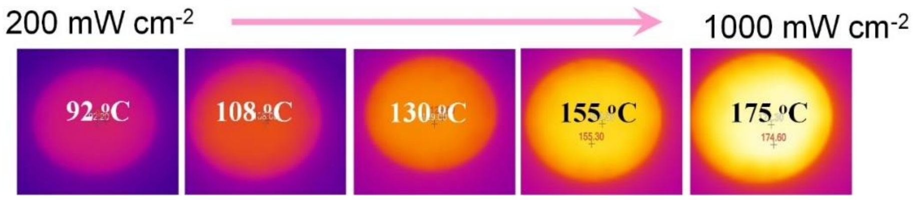

Supplementary Figure 8. Thermal imaging of the surface temperature of catalyst $Ni_{0.66}$-$W_{18}O_{49}$ under AM 1.5G light irradiation with different light intensities (200, 400, 600, 800, and 1000 mW cm^{-2}).

Supplementary Table 3. Comparison between photocatalytic methanol dehydration performance of plasmonic $Ni_{0.66}$-$W_{18}O_{49}$ and the reported thermocatalytic performance in the literatures.

Catalyst	Reaction conditions	Rate (mmol g^{-1} h^{-1})	Methanol conversion (%)	DME Selectivity (%)
ZSM-5	180 °C, 1.1bar	12.9	23	100 [1]
SAPO-11	280 °C	420	80	100 [2]
Al-modified SBA-15-SO_3H	300 °C	53.6	80	100 [3]
6 wt% SiO_2/γ-Al_2O_3	300 °C, 1bar	61.3	85	100 [4]
Alumina pillars modified vermiculite	300 °C, 1bar	20.9	80	98 [5]
$Ni_{0.66}$-$W_{18}O_{49}$ (This work)	400 mW cm^{-2} AM 1.5G light irradiation Batch reactor	133.7 ± 3.3	41.5	99%
$Ni_{0.66}$-$W_{18}O_{49}$ (This work)	1000 mW cm^{-2} AM 1.5 G light irradiation Batch reactor	784.7	94.9	98.5%
$Ni_{0.66}$-$W_{18}O_{49}$ (This work)	Concentrated solar light irradiation (2.3 W cm^{-2}) Continuous reactor	698 ± 50	92.5	99%

Note. In this work, error bars correspond to the standard deviation determined from three independent measurements.

1 Rownaghi, A. A. et al. Selective dehydration of methanol to dimethyl ether on ZSM-5 nanocrystals. *Appl. Catal. B-Environ.* **119**, 56–61 (2012).
2 Chen, Z. et al. Fabrication of nano-sized SAPO-11 crystals with enhanced dehydration of

methanol to dimethyl ether. *Catal. Commun.* **103**, 1–4 (2018).

3 Said, A. E. et al. The catalytic performance of γ-Al_2O_3/red clay as a highly active, selective, and stable catalyst for methanol dehydration to dimethyl ether at competitive low reaction temperature. *Mater. Chem. Phys.* **324**, 129674 (2024)

4 Yaripour, F. et al. Catalytic dehydration of methanol to dimethyl ether (DME) over solid-acid catalysts. *Catal. Commun.* **6**, 147–152 (2005).

5 Marosz, M. et al. Modiffed vermiculites as effective catalysts for dehydration of methanol and ethanol. *Catal. Today* **355**, 466–475 (2020).

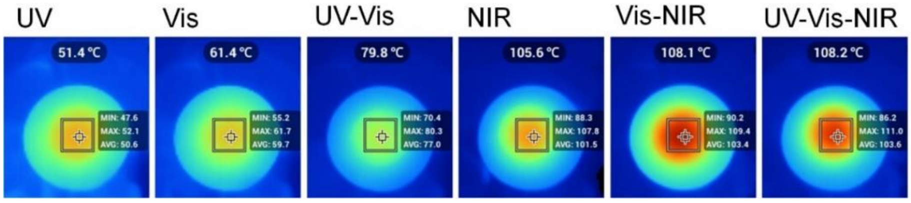

Supplementary Figure 9. Thermal imaging of the surface temperature of catalyst $Ni_{0.66}$-$W_{18}O_{49}$ under different light irradiations (UV, Vis, UV-Vis, NIR, Vis-NIR, and UV-Vis-NIR, with a fixed intensity of 400 mW cm^{-2}).

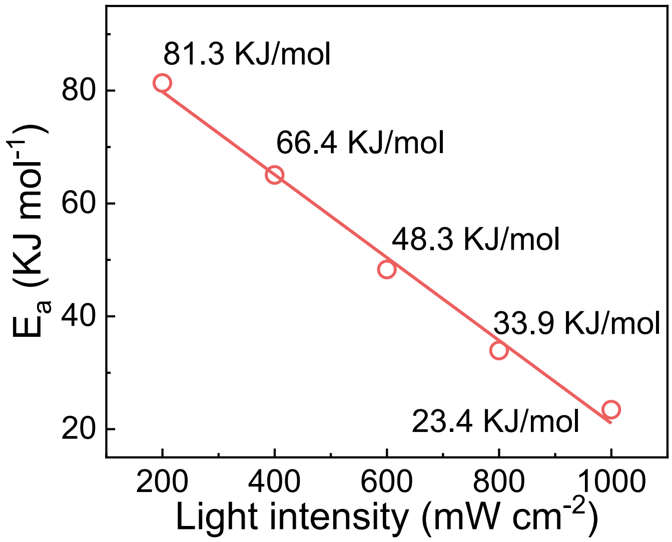

Supplementary Figure 10. The calculated E_a for methanol dehydration reaction over $Ni_{0.66}$-$W_{18}O_{49}$ under AM 1.5G light irradiation with different light intensities (200, 400, 600, 800, and 1000 mW cm^{-2}).

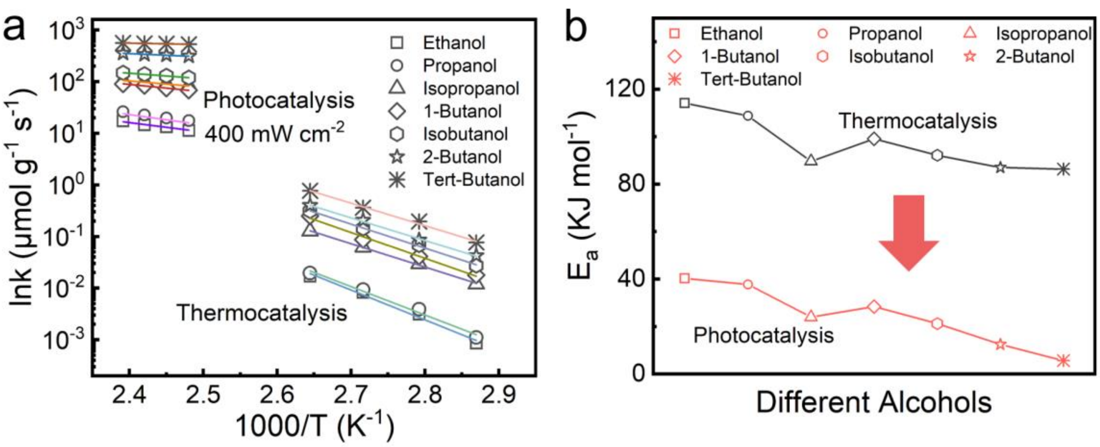

Supplementary Figure 11. **a** Arrhenius plots and **b** the calculated E_a for different alcohol dehydration during thermocatalysis and photocatalysis (400 mW cm^{-2}).

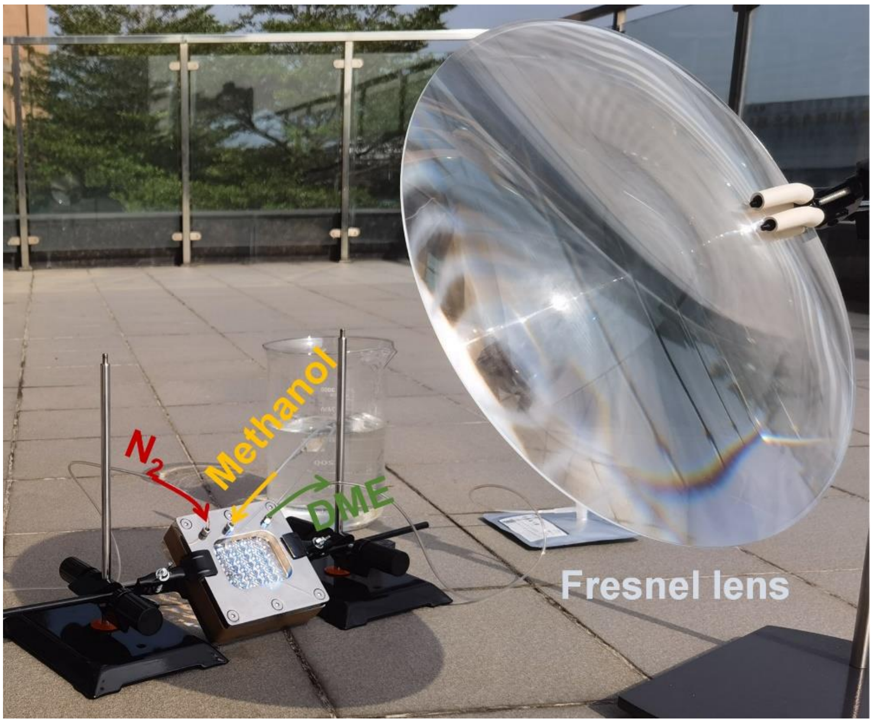

Supplementary Figure 12. The continuous-flow reaction system for photocatalytic methanol dehydration under concentrated solar light irradiation.

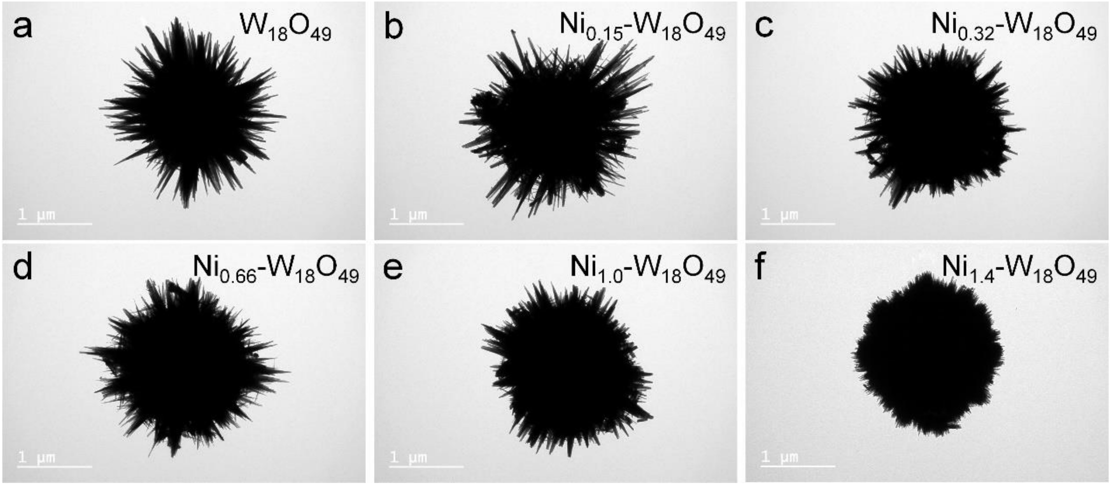

Supplementary Figure 13. TEM images of samples $W_{18}O_{49}$ (a), $Ni_{0.15}$-$W_{18}O_{49}$ (b), $Ni_{0.32}$-$W_{18}O_{49}$ (c), $Ni_{0.66}$-$W_{18}O_{49}$ (d), $Ni_{1.02}$-$W_{18}O_{49}$, (e) and $Ni_{1.4}$-$W_{18}O_{49}$ (f).

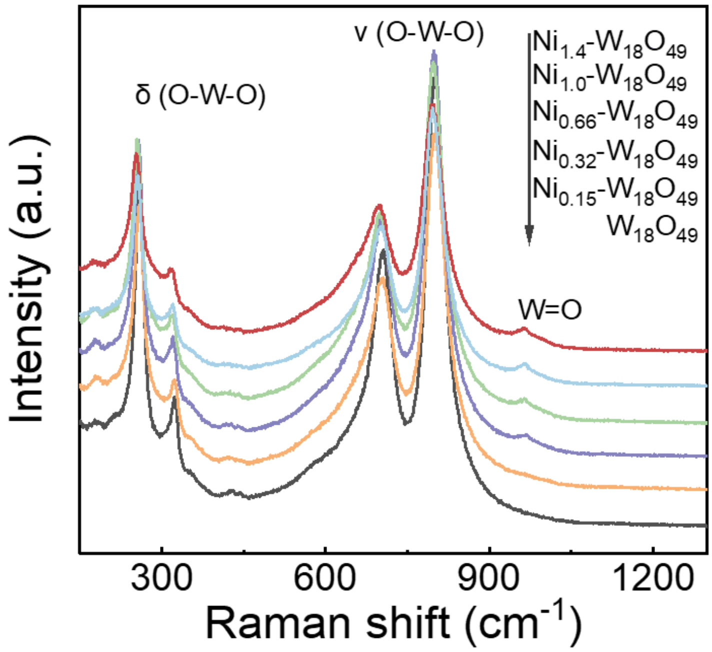

Supplementary Figure 14. Raman spectra of samples $W_{18}O_{49}$ and Ni_x-$W_{18}O_{49}$ (x=0.15, 0.32, 0.66, 1.0, and 1.4).

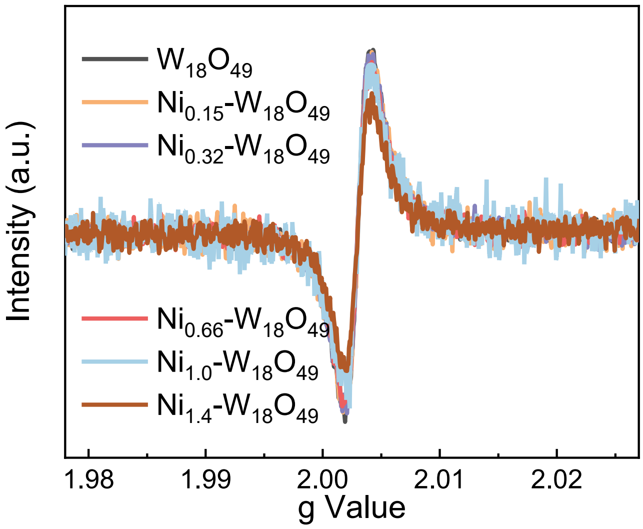

Supplementary Figure 15. EPR spectra of $W_{18}O_{49}$ and Ni_x-$W_{18}O_{49}$ (x=0.15, 0.32, 0.66, 1.0, and 1.4)

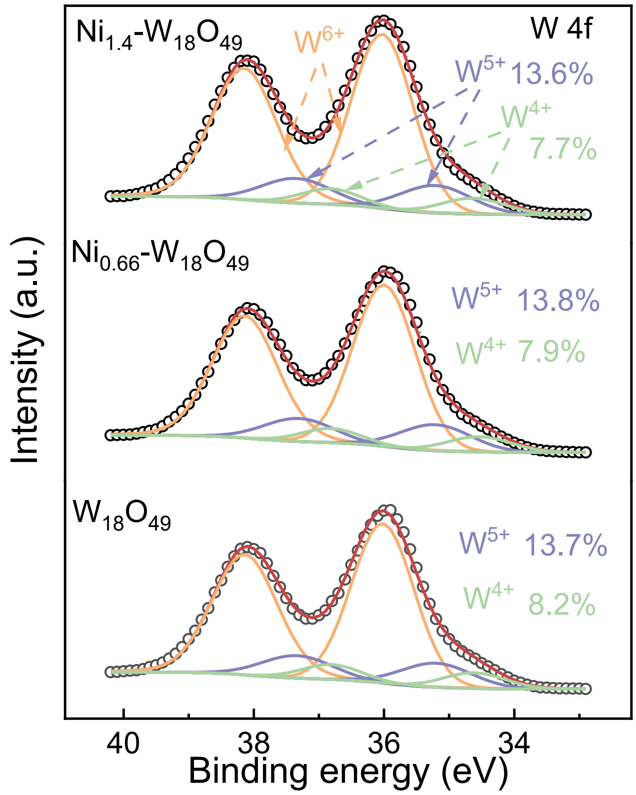

Supplementary Figure 16. XPS of elements W 4f of samples $W_{18}O_{49}$, $Ni_{0.66}$-$W_{18}O_{49}$, and $Ni_{1.4}$-$W_{18}O_{49}$.

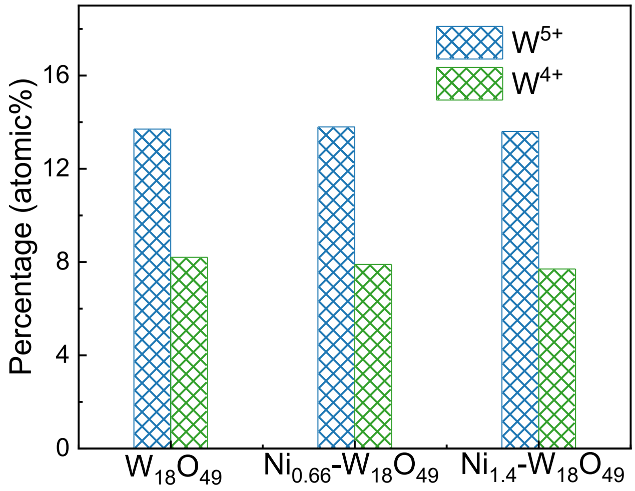

Supplementary Figure 17. Ratio varies of low-valent W (W^{5+}, W^{4+}) in $W_{18}O_{49}$, $Ni_{0.66}$-$W_{18}O_{49}$, and $Ni_{1.4}$-$W_{18}O_{49}$.

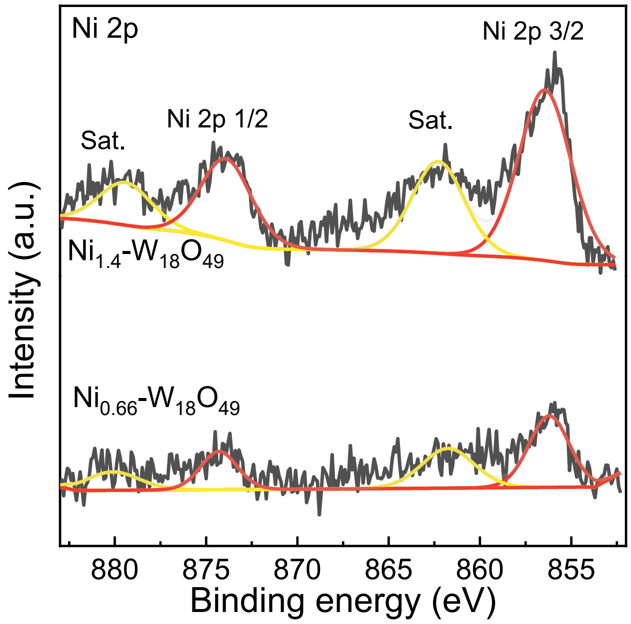

Supplementary Figure 18. XPS of elements Ni 2p of samples $Ni_{0.66}$-$W_{18}O_{49}$ and $Ni_{1.4}$-$W_{18}O_{49}$.

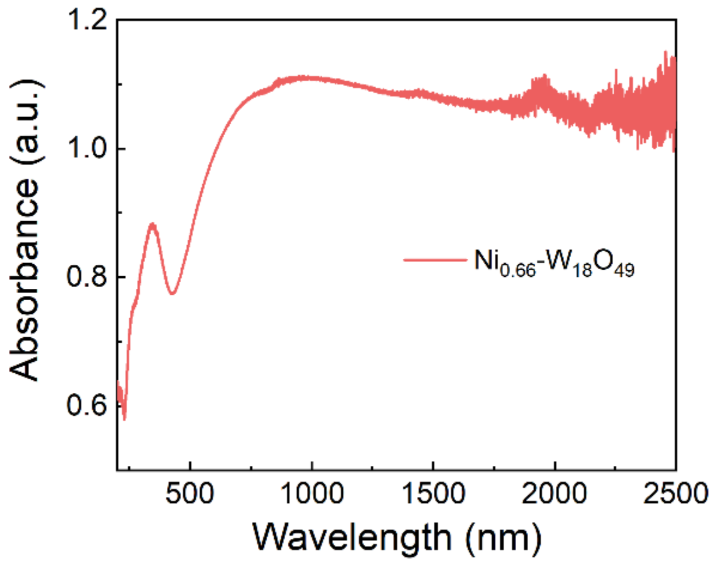

Supplementary Figure 19. UV-Vis-NIR DRS of $Ni_{0.66}$-$W_{18}O_{49}$.

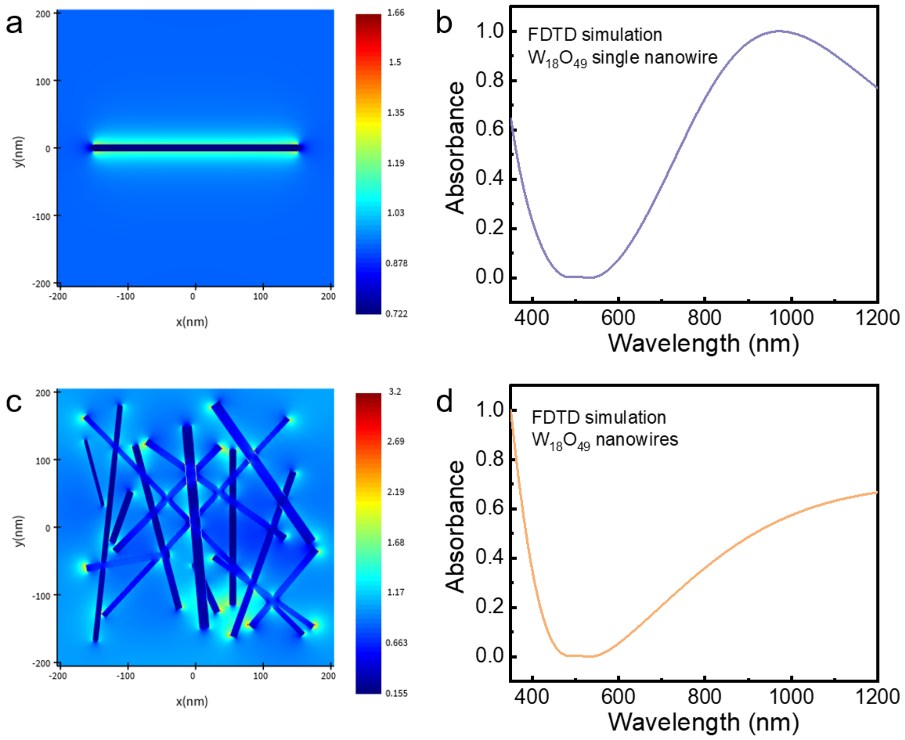

Supplementary Figure 20. Electric field distributions of single $W_{18}O_{49}$ nanowire at 900 nm (a) and stacked $W_{18}O_{49}$ nanowires at 1200 nm (c) presented by FDTD simulations. The light absorption spectra of single $W_{18}O_{49}$ nanowire (b) and stacked $W_{18}O_{49}$ nanowires (d) presented by FDTD simulations.

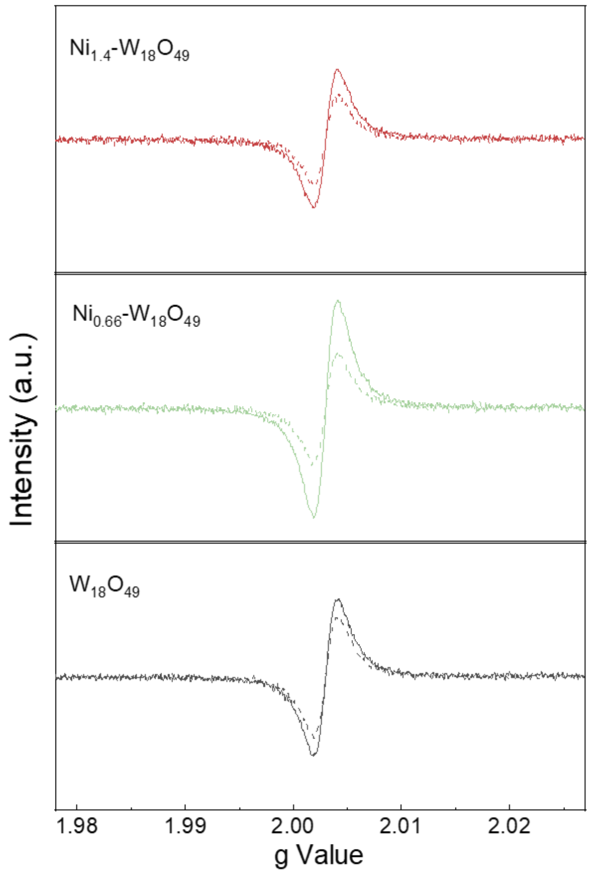

Supplementary Figure 21. EPR spectra of $W_{18}O_{49}$, $Ni_{0.66}$-$W_{18}O_{49}$, and $Ni_{1.4}$-$W_{18}O_{49}$ before (dotted line) and after light irradiation (solid line).

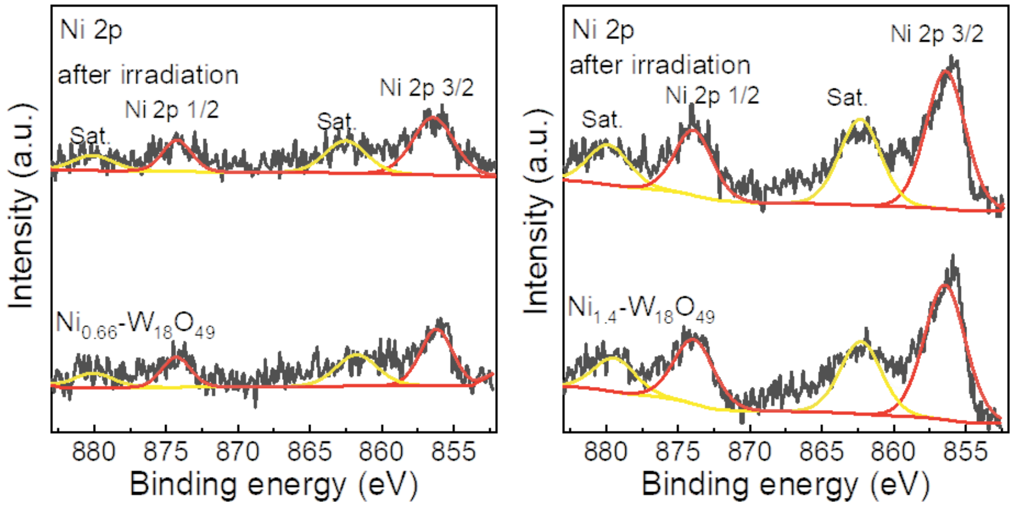

Supplementary Figure 22 XPS of elements Ni 2p of samples $Ni_{0.66}$-$W_{18}O_{49}$ and $Ni_{1.4}$-$W_{18}O_{49}$ before and after light irradiation.

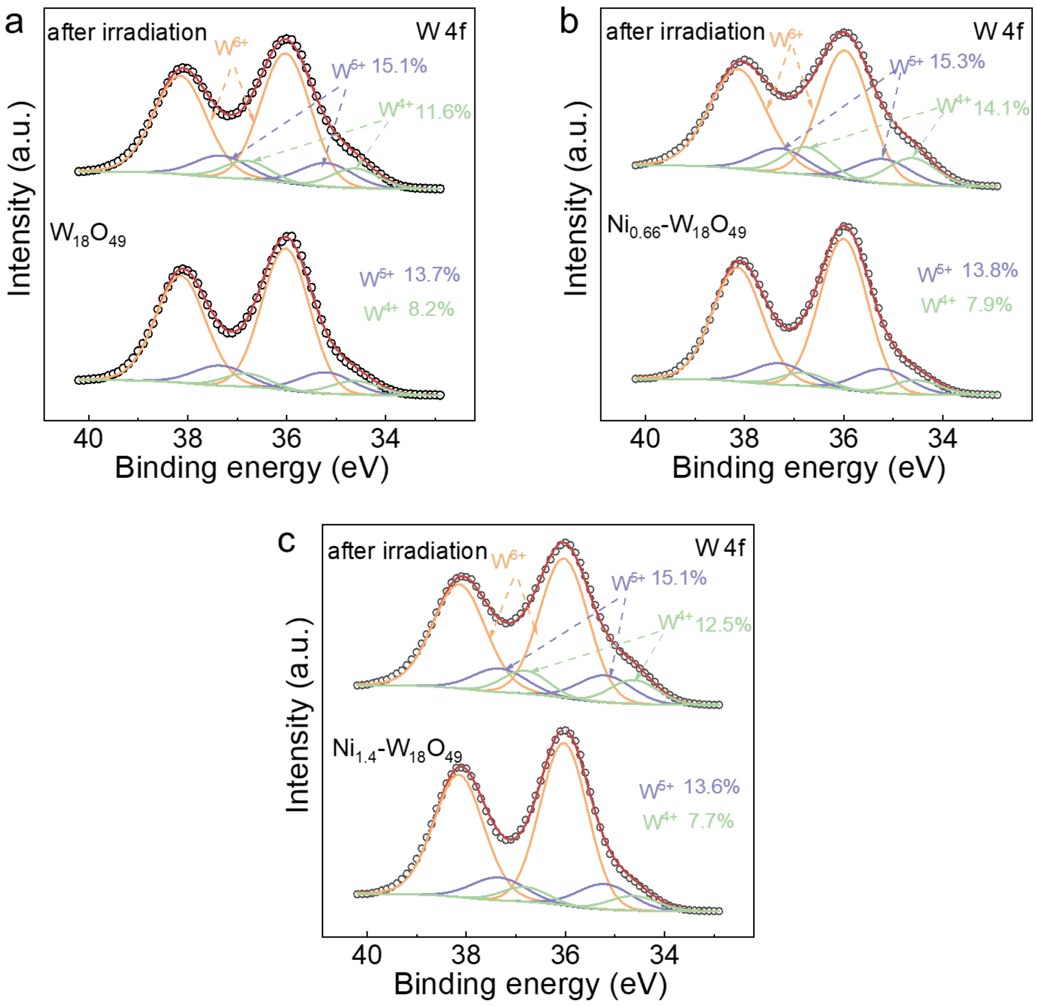

Supplementary Figure 23. W 4f XPS spectra of $W_{18}O_{49}$ (a), $Ni_{0.66}$-$W_{18}O_{49}$ (b) and $Ni_{1.4}$-$W_{18}O_{49}$ (c) before and after light irradiation.

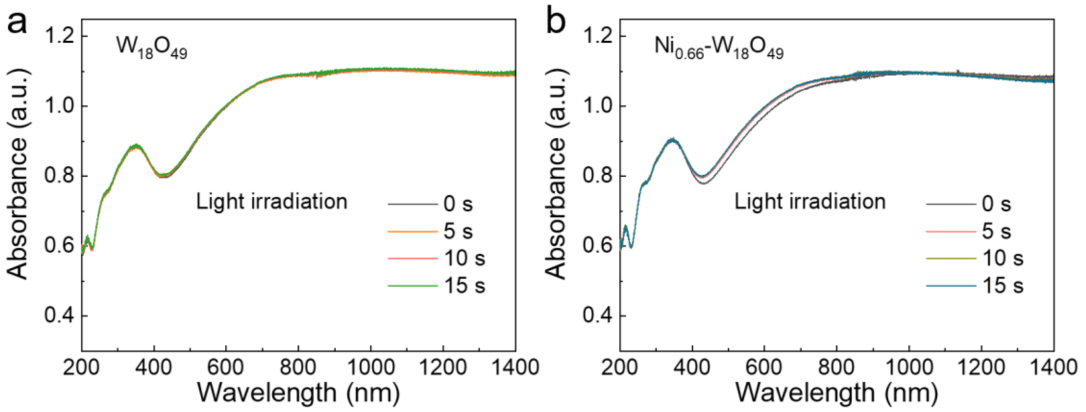

Supplementary Figure 24. UV-Vis-NIR DRS varies of $W_{18}O_{49}$ and $Ni_{0.66}$-$W_{18}O_{49}$ under light irradiation.

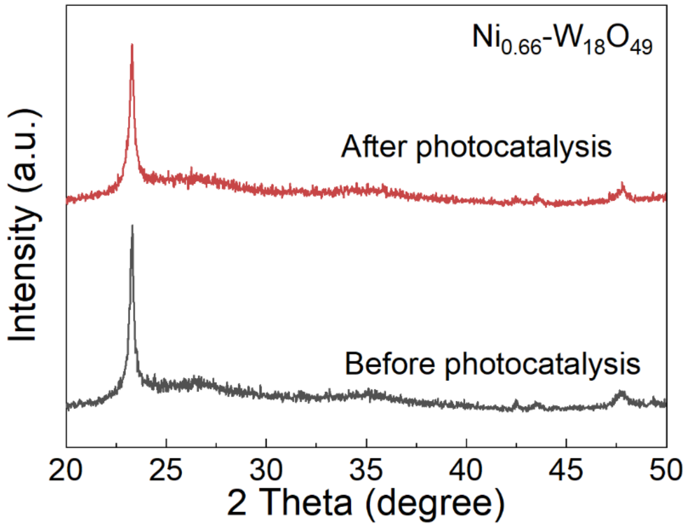

Supplementary Figure 25. XRD patterns of $Ni_{0.66}$-$W_{18}O_{49}$ before and after photocatalysis.

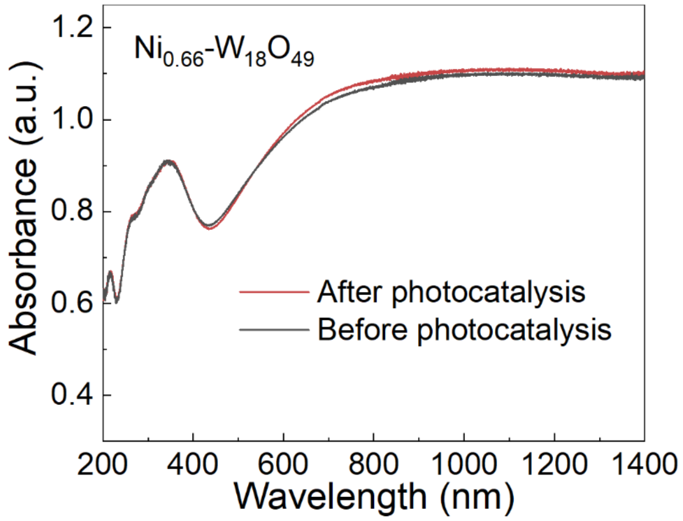

Supplementary Figure 26. DRS of $Ni_{0.66}$-$W_{18}O_{49}$ before and after photocatalysis.

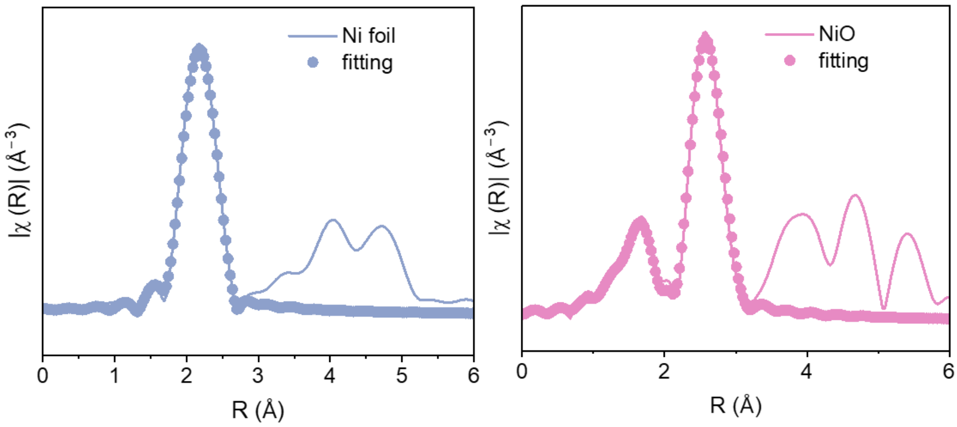

Supplementary Figure 27. The Ni K-edge EXAFS fitting curves for Ni foil and NiO.

Supplementary Table 4. EXAFS fitting parameters at the Ni K-edge for various samples

Sample	Path	CN	R (Å)	σ^2 (Å^2)	ΔE_0 (eV)	R_f
Ni foil	Ni-Ni	12*	2.487	0.007(5)	7.33±0.77	0.008
NiO	Ni-O	6*	2.076	0.007(4)	-2.74±0.60	0.005
	Ni-Ni	12*	2.954	0.006(9)		
$Ni_{0.66}$-$W_{18}O_{49}$	Ni-O	6.37±0.71	2.025	0.006(7)	-5.94±1.28	0.007
$Ni_{1.4}$-$W_{18}O_{49}$	Ni-O	6.30*	2.043	0.011*	-5.25±1.95	0.026
	Ni-Ni	6.19±0.68	3.044			

CN: coordination numbers; *R*: bond distance; σ^2: Debye-Waller factors; ΔE_0: the inner potential correction. R_f: goodness of fit. S_0^2 was fixed to 0.80, according to the experimental EXAFS fit of Ni foil reference by fixing CN as the known crystallographic value.

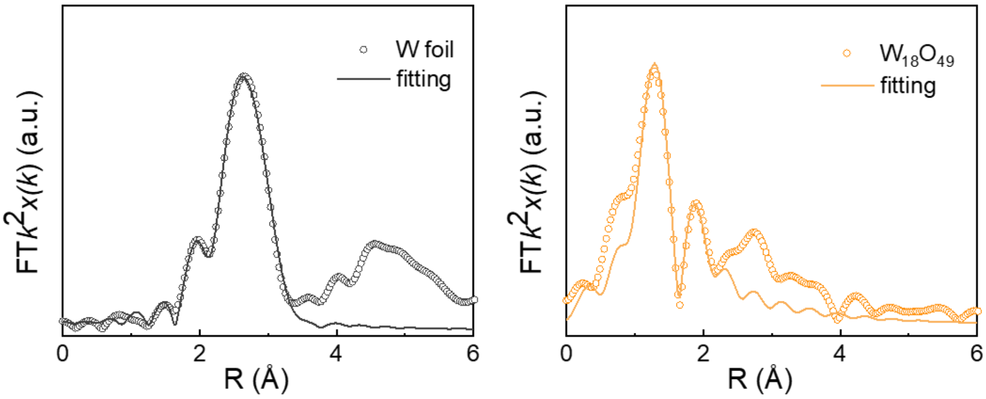

Supplementary Figure 28. The W L3-edge EXAFS fitting curves for W foil and $W_{18}O_{49}$.

Supplementary Table 5. EXAFS fitting parameters at the W L_3-edge for various samples.

Sample	Shell	N^a	R(Å)^b	σ^2(Å^2)^c	ΔE_0 (eV)^d	R factor
W foil	W-W	8*	2.73±0.01	0.0027	5.1±1.9	0.0060
	W-W	6*	3.15±0.02	0.0031		
$W_{18}O_{49}$	W-O1	3.0±0.6	1.79±0.03	0.0031	9.2±6.4	0.0160
	W-O2	3.0±1.1	2.05±0.04	0.0065		
$Ni_{0.66}$-$W_{18}O_{49}$	W-O1	3.0±0.6	1.76±0.03	0.0029	3.7±7.9	0.0199
	W-O2	2.5±1.5	2.00±0.05	0.0073		

$^a N$: coordination numbers; $^b R$: bond distance; $^c\sigma^2$: Debye-Waller factors; ^{d} ΔE_0: the inner potential correction. R factor: goodness of fit. S_0^2 was set to 0.89, according to the experimental EXAFS fit of W foil reference by fixing CN as the known crystallographic value.

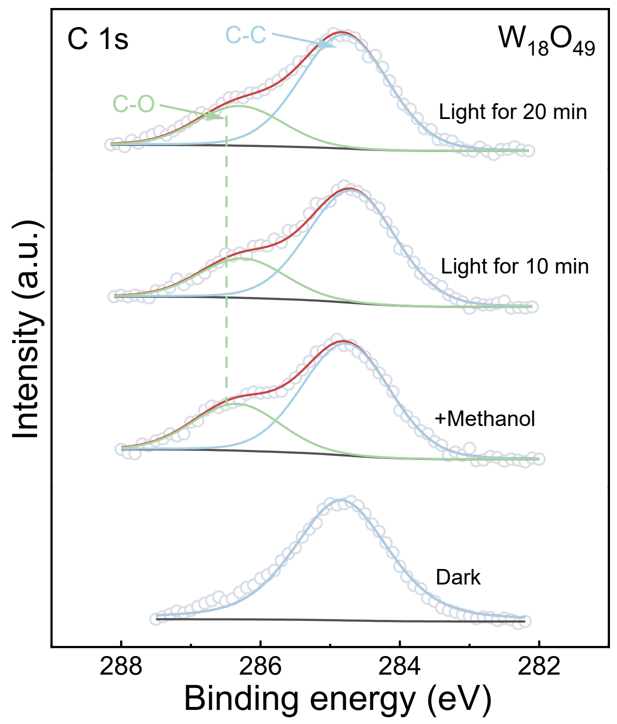

Supplementary Figure 29. *In-situ* C 1s XPS spectra of $W_{18}O_{49}$ during photocatalytic methanol dehydration reaction.

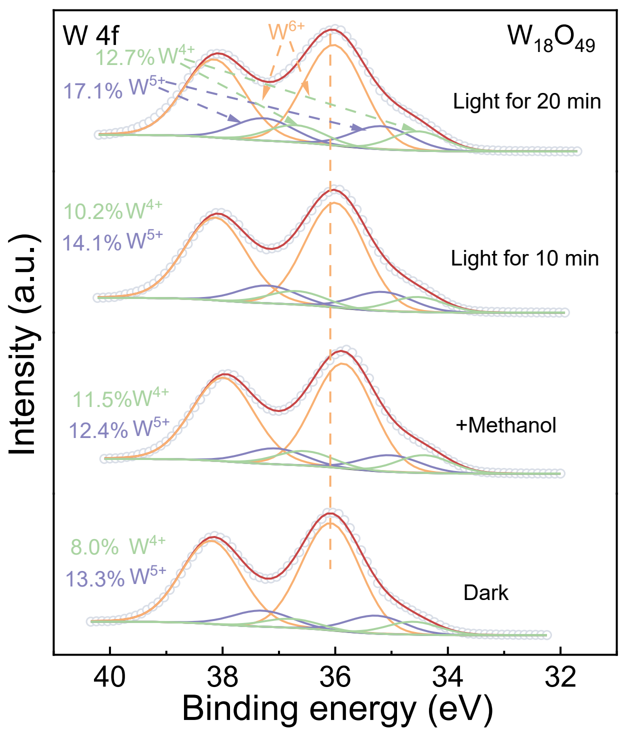

Supplementary Figure 30. *In-situ* W 4f XPS spectra of $W_{18}O_{49}$ during photocatalytic methanol dehydration reaction.

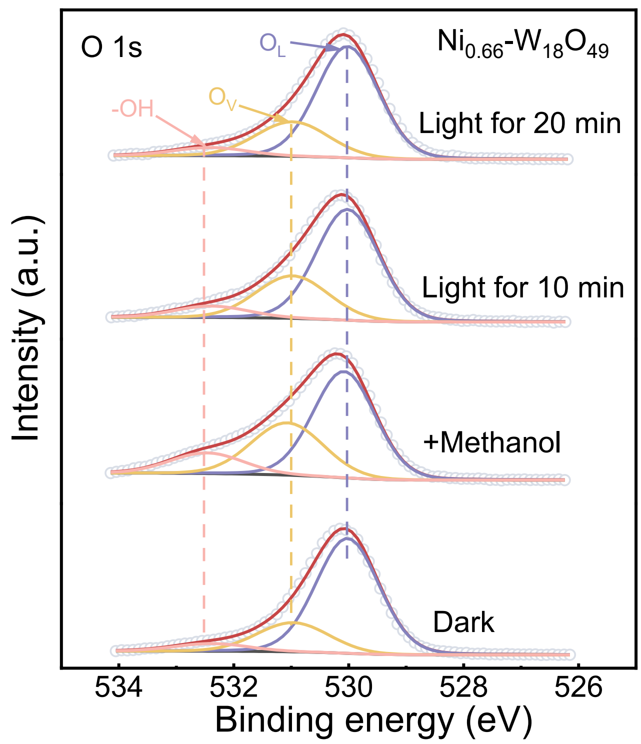

Supplementary Figure 31. *In-situ* O 1s XPS spectra of $Ni_{0.66}$-$W_{18}O_{49}$ during photocatalytic methanol dehydration reaction.

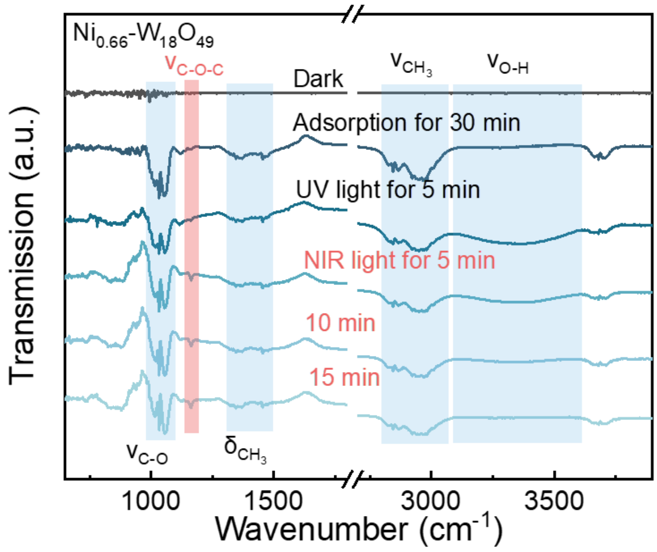

Supplementary Figure 32. *In-situ* FT-IR transmission spectra of $Ni_{0.66}$-$W_{18}O_{49}$ during photocatalytic methanol dehydration reaction under UV irradiation for 5 min, and then NIR irradiation for 15 min.

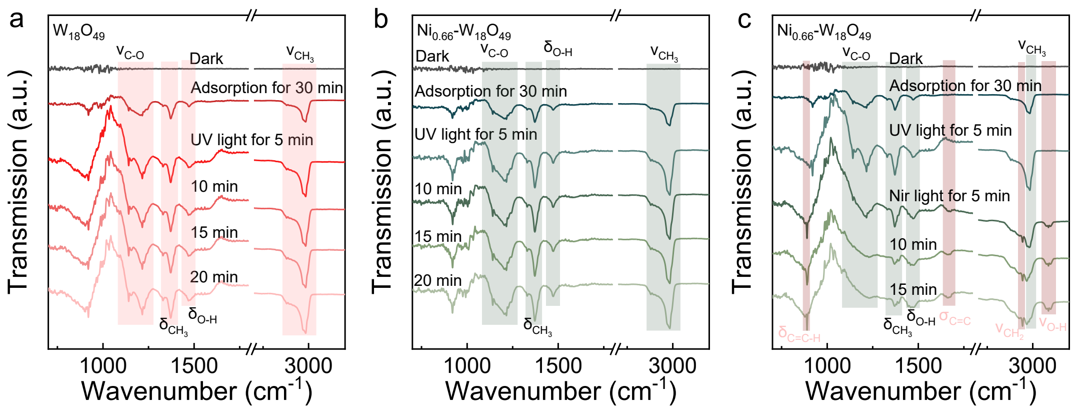

Supplementary Figure 33. In-situ FT-IR spectroscopy of (a) $W_{18}O_{49}$ and (b) $Ni_{0.66}$-$W_{18}O_{49}$ during photocatalytic tert-butanol dehydration reaction under UV light irradiation, and (c) $Ni_{0.66}$-$W_{18}O_{49}$ under UV for 5 min and NIR for 15 min.

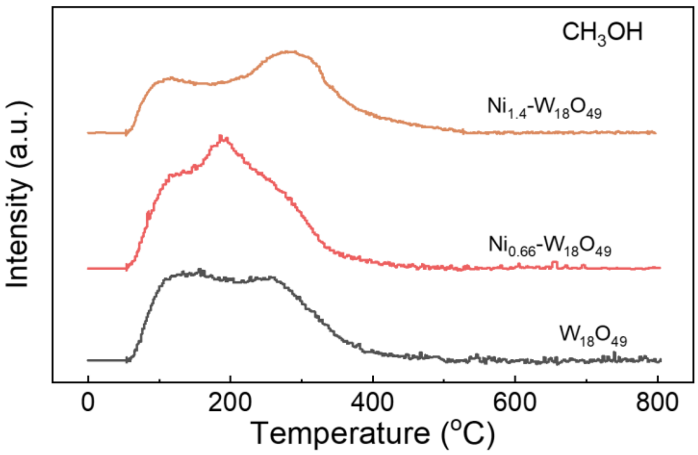

Supplementary Figure 34. The methanol-TPD pattern of the $W_{18}O_{49}$, $Ni_{0.66}$-$W_{18}O_{49}$ and $Ni_{1.4}$-$W_{18}O_{49}$ catalysts.

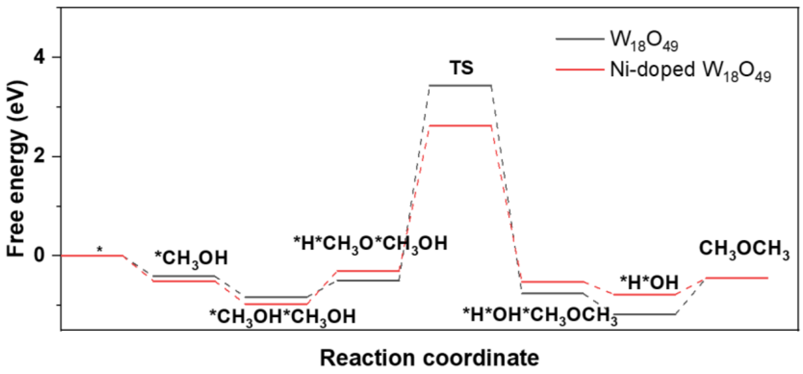

Supplementary Figure 35. DFT calculated energy diagram of methanol dehydration reaction over $W_{18}O_{49}$ and Ni-doped $W_{18}O_{49}$.

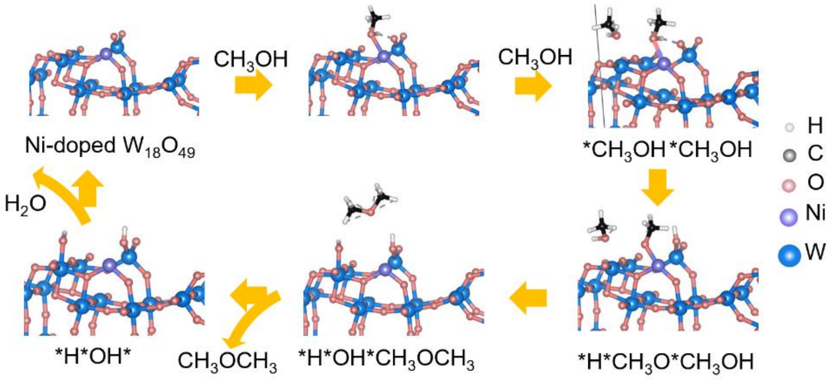

Supplementary Figure 36. DFT simulations on reaction pathway of methanol dehydration over Ni-doped $W_{18}O_{49}$.

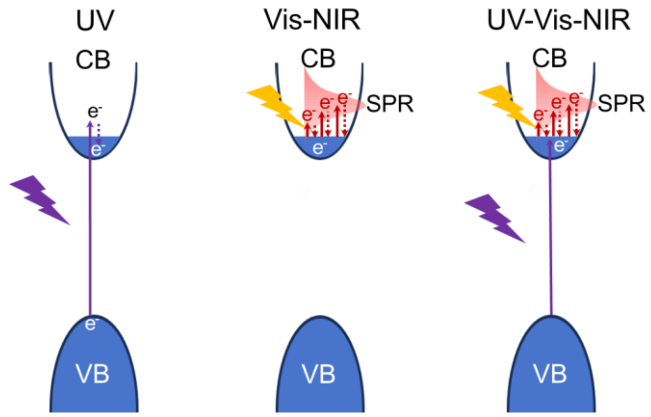

Supplementary Figure 37. The excitation processes on $Ni_{0.66}$-$W_{18}O_{49}$ under UV, Vis-NIR and UV-Vis-NIR light irradiations.

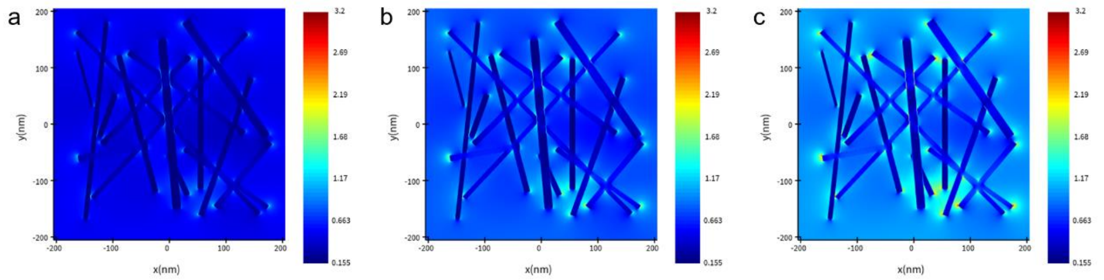

Supplementary Figure 38. Electric field distributions of stacked $W_{18}O_{49}$ nanowires at UV (350 nm, a), Vis (650 nm, b), and NIR (1200 nm, c) simulated by FDTD calculations.
