# Supplementary material for: Plasmonic Ni-doped W18O49 with dual active sites drives efficient methanol dehydration to dimethyl ether
Source: Nat Commun. 2025 Nov 17;16:10062. doi: 10.1038/s41467-025-65040-3 (PMC12623422; doi:10.1038/s41467-025-65040-3)
Supplement: Supplementary file 2 — Description of Addtional Supplementary File [file 41467_2025_65040_MOESM2_ESM.pdf]

Description of Additional Supplementary File

Supplementary Data 1: contains two files, which are the DFT models of pristine $W_{18}O_{49}$ and Ni-doped $W_{18}O_{49}$, respectively.
